# Oxytocin pathway gene variation and corticostriatal resting-state functional connectivity

**DOI:** 10.1016/j.cpnec.2024.100255

**Published:** 2024-07-31

**Authors:** Shanshan Xiao, Håkan Fischer, Natalie C. Ebner, Gull Rukh, Junhua Dang, Lars Westberg, Helgi B. Schiöth

**Affiliations:** a Department of Psychology, Stockholm University, Campus Albano house 4, Albanovägen, SE-114 19, Stockholm, Sweden; b Department of Surgical Sciences, Functional Pharmacology and Neuroscience, Uppsala University, BMC, Husargatan 3, 75124, Uppsala, Sweden; c Stockholm University Brain Imaging Centre (SUBIC), SE-106 91, Stockholm, Sweden; d Aging Research Center, Karolinska Institutet and Stockholm University, Tomtebodavägen 18 A, SE-171 77, Stockholm, Sweden; e Department of Psychology, University of Florida, P.O. Box 112250, Gainesville, FL, 32611-2250, USA; f Cognitive Aging and Memory Program, Clinical Translational Research Program (CAM-CTRP), University of Florida, 2004 Mowry Road, Gainesville, FL, 32611, USA; g McKnight Brain Institute, University of Florida, 1149 Newell Drive Gainesville, FL, 32610, USA; h Institute of Social Psychology, School of Humanities and Social Sciences, Xi'an Jiaotong University, No.28 Xianning West Road, Xi'an, Shaanxi, 710049, PR China; i Department of Pharmacology, Institute of Neuroscience and Physiology at the Sahlgrenska Academy, University of Gothenburg, Box 431, SE-405 30, Gothenburg, Sweden

**Keywords:** Oxytocin pathway gene, Corticostriatal connectivity, Resting-state functional connectivity, UK Biobank

## Abstract

Genetic variations in single nucleotide polymorphisms (SNPs) within oxytocin pathway genes have been linked to social behavior and neurodevelopmental conditions. However, the neurobiological mechanisms underlying these associations remain elusive. In this study, we investigated the relationship between variations of 10 SNPs in oxytocin pathway genes and resting-state functional connectivity among 55 independent components using a large sample from the UK Biobank (N ≈ 30,000). Our findings revealed that individuals with the GG genotype at rs4813627 within the oxytocin structural gene (*OXT*) exhibited weaker resting-state functional connectivity in the corticostriatal circuit compared to those with the GA/AA genotypes. Empirical evidence has linked the GG genotype at *OXT* rs4813627 with a behavioral tendency of insensitivity to others. These results inform the neural mechanisms by which oxytocin-related genetic factors can influence social behavior.

## Highlights

• Oxytocin may affect brain regions enriched with oxytocin pathway genes expression, indirectly affecting other regions via neural connections.
• *OXT* rs4813627 variations were associated with resting-state functional connectivity within the corticostriatal circuit in a UK biobank sample.
• *OXT* rs4813627 GG carriers showed weaker corticostriatal connectivity compared to allele A carriers (GG/AA genotypes).

## 1 Introduction

Oxytocin is a neuropeptide that plays a crucial role in human physiology and social behavior. It can enhance social sensitivity, promote social information processing, facilitate social behavior such as mother-infant bonding, cooperation, and group affiliation [1,2], and has been linked with neurodevelopmental conditions such as autism spectrum disorder (ASD) that are characterized by social deficits [3]. There is a pathway along which oxytocin is generated and transported to act on the neural system. First, oxytocin is primarily synthesized in neurons located in the supraoptic and paraventricular nuclei of the hypothalamus [4]. It is then transported to several forebrain regions for central release, and to the posterior pituitary for peripheral release [5,6]. Locally, oxytocin exerts its effects by binding to oxytocin receptors distributed throughout the brain and the periphery [[7], [8], [9]].

There are various genes regulating these functions, of which three have received the most attention in the literature to date: the oxytocin/neurophysin I prepropetide (*OXT*) gene, the cluster of differentiation 38 glycoprotein (*CD38*) gene, and the oxytocin receptor (*OXTR*) gene. Each of these genes plays a unique role, with the *OXT* as the structural gene for oxytocin, encoding a precursor protein that produces oxytocin and neurophysin I [10,11], the *CD38* regulating oxytocin release [12], and the *OXTR* determining density and availability of oxytocin receptor sites [13,14].

These oxytocin pathway genes contain numerous single nucleotide polymorphisms (SNPs). Genetic variations in these SNPs have been associated with social behavior and neurodevelopmental conditions [15,16]. For instance, a meta-analysis has indicated associations between ASD and the SNPs rs7632287, rs237887, rs2268491, and rs2254298 in *OXTR* [17]. However, the neurobiological processes underlying these associations are not well understood. Researchers have suggested that central release of oxytocin may rewire neural networks by strengthening (or weakening) connections between neurons and thus altering the way neuronal networks function [18]. This rewiring may originate from brain regions enriched with expression of oxytocin pathway genes [[19], [20], [21], [22]]. That is, oxytocin may directly influence activity of brain regions enriched with expression of oxytocin pathway genes. These brain regions are intrinsically connected with other brain regions, and activity of oxytocin-enriched regions spreads to other brain regions via these intrinsic neural connections. This conjecture can be tested by confirming connectivity between regions enriched with expression of oxytocin pathway genes and other regions in the brain. For instance, individuals with the GG genotype at rs3796863 in *CD38*, compared to T allele carriers, show enhanced resting-state connectivity between the ventral striatum (an area with high oxytocin pathway gene expression) and cingulate cortex, which may contribute to their increased alcohol self-administration [23].

The current study set out to examine the conjecture mentioned above in the UK Biobank. In particular, the UK Biobank has performed group independent component analysis (ICA) parcellation on resting-state fMRI data, with 55 spatially independent components extracted, each of which covers several non-contiguous regions with correlated time courses [24,25]. The database also contains resting-state functional connectivity between each pair of these 55 components. For the analysis in this study, we focused on functional connectivity at rest between the ICA component that included most of the brain regions enriched with expression of oxytocin pathway genes and all other 54 ICA components identified in the UK Biobank (see Methods and Materials for details). We further examined the moderation of SNPs in oxytocin pathway genes on resting-state functional connectivity, which enabled us to determine how inter-subject genetic variability in these oxytocin pathway genes would affect associated functional connectivity at rest.

Although researchers have begun using imaging genetics to explore the impact of genetic variations within oxytocin pathway genes on brain function [16,26], the bulk of these studies have focused primarily on *OXTR* SNPs. Few studies have given attention to *CD38* and *OXT* SNPs, not to mention examining all three oxytocin pathway genes across the same set of participants [16]. Furthermore, sample sizes in these previous studies were small, limiting statistical power and obfuscating true relationships between generic variations and functional brain connectivity. Going beyond previous works, we considered important SNPs within all three oxytocin pathway genes (i.e., *OXT*, *CD38*, and *OXTR*) [15,26,27], thus capturing function pertaining to oxytocin's multiple mechanisms of release, transmission, and binding [28,29]. Meanwhile, the UK Biobank offers a dataset of over 30,000 participants with genetic and brain imaging data, which is 100-fold larger than any dataset used in prior studies and provides sufficient power to detect small effects of single SNPs within oxytocin pathway genes.

## 2 Methods and Materials

This study incorporated genetic and neuroimaging data from the UK Biobank [30], authorized by the National Health Service Research Ethics committees (No. 11/NW/0382). Informed written consent was obtained from all participants prior to data collection. The study was conducted using the UK Biobank resource under Application Number 30172. The ethical application for using the data was approved by the Regional Ethics Review Board in Uppsala (Dnr 2017/198).

### 2.1 Participants

The UK Biobank is a comprehensive biomedical database that enrolled over 500,000 participants throughout the United Kingdom between 2006 and 2014. It contains extensive measures collecting genetic, health, and cognitive information [25,[30], [31], [32]]. Between 2016 and 2022, a subset of the initial participants underwent multimodal brain imaging [25]. At the time of this study, resting-state data obtained from the initial MRI scan of 39,867 participants were available for analysis.

As the present study investigated the association between variations in oxytocin pathway genes and resting-state functional connectivity, participants needed to have both resting-state and genetic data for inclusion. Because not all participants had data for the complete set of SNPs of interest (e.g., some participants had missing data for one or more SNPs), the number of individuals with both resting-state fMRI data and genetic data varied for each SNP. Furthermore, individuals who did not self-report as white British (Data-Field 21000, “Ethnic background”) or had more than 5 % of genotypes missing (Data-Field 22005, “Missingness”) were excluded from the sample. More detailed information is provided in Table 1.

**Table 1:** Demographic and genotype information of the 10 SNPs included in the final analysis.

Gene	SNP	Major allele	Minor allele	Minor allele frequency (MAF)	Subjects with 0 minor allele	Subjects with 1 minor allele	Subjects with 2 minor alleles	Total sample	Age (M ± SD, in years)	Sex (% females)
*OXT*	rs4813627	A	G	0.48	8422	15533	7022	30977	64.14 ± 7.65	52.65 %
*OXT*	rs4813625	G	C	0.47	7339	13064	5968	26371	64.15 ± 7.65	52.88 %
*CD38*	rs3796863	C	A	0.30	17552	14731	3151	35434	64.16 ± 7.64	52.70 %
*OXTR*	rs11914885	A	G	0.30	17515	15512	3323	36350	64.16 ± 7.64	52.65 %
*OXTR*	rs7632287	G	A	0.25	20286	13826	2238	36350	64.16 ± 7.64	52.65 %
*OXTR*	rs237887	A	G	0.42	12212	17741	6397	36350	64.16 ± 7.64	52.65 %
*OXTR*	rs7634632	T	C	0.49	9350	18032	8640	36022	64.15 ± 7.64	52.68 %
*OXTR*	rs237851	G	A	0.46	10486	17509	7562	35557	64.16 ± 7.65	52.72 %
*OXTR*	rs2268498	T	C	0.45	10476	17271	7179	34926	64.15 ± 7.64	52.72 %
*OXTR*	rs53576	G	A	0.31	15759	14292	3273	33324	64.14 ± 7.65	52.73 %

Abbreviations: M = Means, SD = Standard Deviations.

With more than 30,000 participants with genetic and brain imaging data, the UK Biobank offers a dataset that is 100-fold larger than any used in prior studies. A recent meta-analysis assessed the effects of *OXTR* rs53576 on empathy and found that the GG genotype showed greater affective empathy than other genotypes in European cohorts [33], with an effect size of Cohen's *d* = 0.12. This effect size equals *f* = 0.06 in a one-way ANOVA as applied in the present paper (see details regarding our analytic approach below). Based on this prior work, we conducted a sensitivity analysis using the software G*Power. Results from this analysis indicated that our sample size could detect an effect size of *f* = 0.02 with a power of 0.99 at the significance level of 0.05 in a one-way ANOVA comprising three genotypes. Thus, our sample had sufficient statistical power to detect small effects of single SNPs within oxytocin pathway genes.

### 2.2 Imaging data

To analyze resting-state functional connectivity, we employed network matrices produced through an image-processing pipeline developed and executed by the UK Biobank Imaging Project team [24]. Below is a concise description of the image acquisition protocols and processing pipelines. More details can be found in a previous article [25].

Brain imaging data were obtained on a Siemens Skyra 3T Magnetic Resonance Imaging (MRI) Scanner (Siemens Medical Solutions, Erlangen, Germany; see https://biobank.ctsu.ox.ac.uk/crystal/refer.cgi?id=2367) equipped with a 32-channel receive head coil. Participants were positioned supine during the imaging process, instructed to relax, and fixate their gaze upon a crosshair.

The UK Biobank performed preprocessing, group-ICA parcellation, and connectivity estimation of the data using FSL packages [25]. The raw data underwent standard preprocessing procedures, including motion correction with MCFLIRT, normalization of grand-mean intensity with a single multiplicative factor, high-pass temporal filtering through Gaussian-weighted least-squares straight line fitting (sigma was set to 50.0 s), EPI unwarping using a pre-collection field map, gradient distortion correction unwarping, and removal of structural artifacts with an ICA-based X-noiseifier. To ensure data quality, any noticeable preprocessing errors were checked and removed through visual inspection [25].

Resting-state networks can be identified using ICA [34,35], a method that detects spatially independent components within the data, where each component consists of a spatial map and a single associated time course. In the UK Biobank project, group-ICAs in FSL packages were used to parcellate the dataset into two different dimensionalities (100 and 25 spatially independent components) on a preprocessed sample of 4162 individuals. After removing noise components, two sets of 55 and 21 neuronally driven components were obtained. The 55 components provided a parcellation with higher spatial resolution, meaning that distinct regions within these spatial component maps were smaller and more detailed. In contrast, the low-resolution parcellation of the 21 components enabled the estimation of temporal synchronization between bulk networks, resulting in a large-scale network decomposition that matches much of the existing literature on resting-state networks [24,25,36]. The maps for both ICAs can be found at https://www.fmrib.ox.ac.uk/datasets/ukbiobank/.

For the current study, the 55-component decomposition was used because it provides a parcellation with higher spatial resolution. The ICA spatial maps were mapped onto the resting-state fMRI time series for each subject to obtain one representative time series per ICA component (each of which was considered a network “node”). Node time series were then used to estimate subject-specific network matrices. Network modeling was performed using the FSLNets toolbox for full and partial temporal correlation coefficients between each pair of node time series, resulting in a 55 × 55 matrix of connectivity estimates.

In this study, we used partial correlation matrices generated by controlling for the strength of other connections, thus obtaining more accurate estimates of direct connection strengths than those achieved by full correlation. Normalized estimation of partial correlation was conducted with L2 regularization applied (setting rho = 0.5 in the Ridge Regression option in FSLNets) to slightly enhance partial correlation coefficient estimates. Network matrix values were then transformed from Pearson correlation scores (r-values) to z-statistics [24,25]. The resulting 55 × 55 partial correlation matrices were utilized to measure resting-state functional connectivity.

### 2.3 Resting-state functional connectivity of interest

**Fig. 1:**
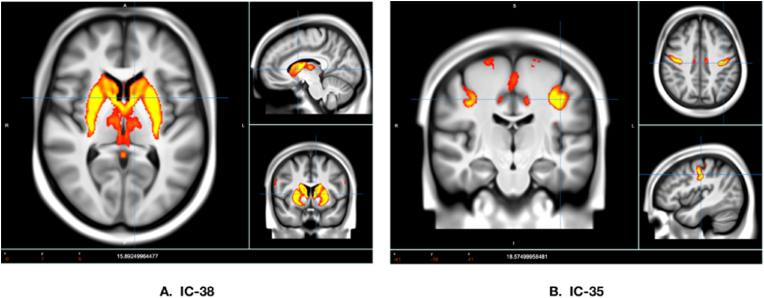
Spatial maps of **(A)** component IC-38 and **(B)** component IC-35. Voxels are color-coded based on Z-statistics, which indicate the degree of fit between the time series of each voxel and the component's time series. (For interpretation of the references to color in this figure legend, the reader is referred to the Web version of this article.)

The expression of oxytocin pathway genes is not evenly distributed across the brain. By creating voxel-by-voxel volumetric expression maps for the distribution of *OXT*, *CD38*, and *OXTR* mRNA, Quintana et al. [37] identified a group of subcortical regions (e.g., thalamus, caudate, pallidum, and putamen) that are enriched with the expression of the three selected oxytocin pathway genes. Among the 55 ICA components, the independent component 38 (IC-38) was of particular interest in this study as it contained brain regions with high expression of oxytocin pathway genes (see Fig. 1A). Thus, we focused on functional connectivity between IC-38 and the other 54 components, which allowed us to test whether genetic variations within oxytocin pathway genes affected functional connectivity between regions enriched with expression of oxytocin pathway genes and other regions in the brain.

### 2.4 SNP selection and genotyping

Prior to data analysis, we implemented rigorous quality control on the genetic data to ensure robustness of the findings. A set of 28 oxytocin-related SNPs was identified based on their associations with social behaviors revealed by previous review articles [15,26,27]. However, of those 28 SNPs, only 20 were available in the UK Biobank dataset. These included rs4813627 and rs4813625 for *OXT*; rs3796863 for *CD38*; and rs53576, rs7634632, rs11914885, rs237851, rs237887, rs2268490, rs2268493, rs2268494, rs2268498, rs4686302, rs4564970, rs2301261, rs7632287, rs75775, rs6770632, rs2740210, and rs6449197 for *OXTR*. Of these 20 SNPs, 3 with minor allele frequencies (MAF) < 10 % (rs2268494, rs2301261, rs4564970) were excluded from our analysis; and another 7 SNPs (rs2740210, rs6449197, rs6770632, rs2268490, rs2268493, rs4686302, rs75775) having high linkage disequilibrium (D' > 0.8, we retained the one that was highlighted in previous studies) with other SNPs in the sample were excluded as well. We also evaluated the genotype distribution for each SNP to confirm no deviation from the expected values under the Hardy-Weinberg equilibrium. No SNPs were excluded based on the p > .05 threshold. Details regarding genotype information for the final set of 10 SNPs analyzed here are summarized in Table 1.

### 2.5 Statistical analysis

Our analyses were organized around the conjecture that oxytocin influences the activity of brain regions enriched with expression of oxytocin pathway genes, and such activity would then spread to other brain regions via neural connections. This conjecture can be examined by testing functional connectivity of regions enriched with expression of oxytocin pathway genes with other regions in the brain. The UK Biobank identified 55 independent components based on group-ICA, among which IC-38 contained brain regions with high expression of oxytocin pathway genes. Our study determined the effects of variations in oxytocin pathway genes on functional connectivity of IC-38 with the other 54 ICA components.

To address such effects, the following statistical procedures were performed. First, we conducted a series of one-way ANOVAs, one for each of the 10 SNPs listed in Table 1 (categorizing participants into three different genotypes such as AA carriers, GA carriers, and GG carriers) as the independent variable and functional connectivity between IC-38 and each of the other 54 ICA components as the dependent variable, respectively. In each of these ANOVAs, SNP was treated as a fixed factor, and sex and age were controlled by incorporating them as random factors [38]. Additionally, to manage potential population stratification, the first ten genetic principal components (Data Field 22009) were included as covariates in each ANOVA. Statistical analyses were performed in RStudio (software version 4.2.1) using the afex package (version 1.2.1) for ANOVAs. Second, given there were 10 SNPs and 54 functional connections, a total of 540 ANOVAs were performed. We therefore applied multiple comparison correction (*p* < .05, two-tailed) to reduce the Type 1 error. Finally, post-hoc comparisons were carried out using the emmeans package (version 1.8.1.1) to determine differences among genotypes categorized by SNPs that survived multiple comparison correction.

Finally, to test the robustness of our findings, we conducted an analysis using cross-validation [39]. This method employs two metrics to evaluate how well the proposed model fits the data: *R*^2^ and root mean square error (*RMSE*). *R*^2^ indicates the proportion of the variance in the outcome variable explained by the predictors in the model, whereas *RMSE* is defined as the square root of the average of the squared differences between expected and observed values, indicating the deviation of expected values from observed values. First, we calculated *R*^2^ and *RMSE* from the full dataset. Then, we performed k-fold cross-validation, splitting the full dataset into 10 equally sized folds. The proposed model was trained on 9 folds and tested on the remaining fold. This process was repeated 10 times, with each fold serving as the test set once. If the two metrics varied slightly across the 10 iterations and their averages over the 10 iterations were comparable to the *R*^2^ and *RMSE* resulting from the full dataset, it demonstrates that the proposed model is robust.

## 3 Results

**Fig. 2:**
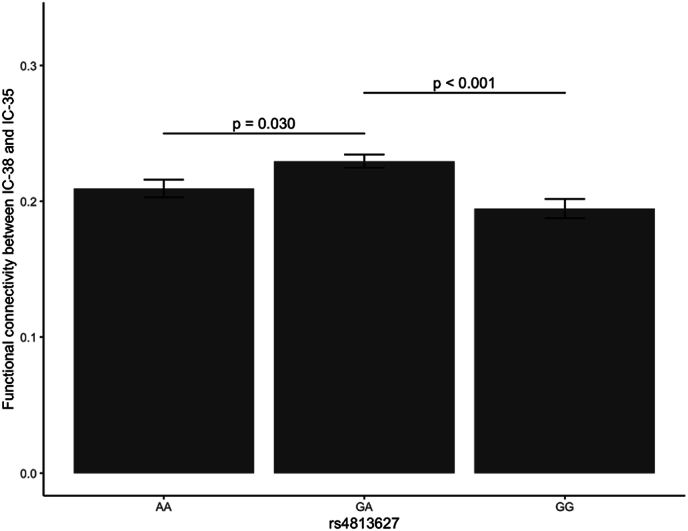
Resting-state functional connectivity between IC-38 and IC-35 for the three genotypes of the *OXT* rs4813627 SNP. Post-hoc testing revealed that GG and AA carriers exhibit weaker corticostriatal functional connectivity between IC-38 and IC-35 compared to GA carriers. Error bars denote the Standard Error (SE).

First, as shown in Table 2, there were 33 significant one-way ANOVA results (for the complete list of 540 ANOVAs, see supplementary information). Second, after multiple comparison correction, only one pair of components, IC-38 and IC-35 (Fig. 1A and B), survived. Although there were other significant results (i.e., differences between variations in other SNPs regarding functional connectivity of IC-38 with some of the other 54 components), they did not survive Bonferroni correction. Specifically, the effect of variations in *OXT* rs4813627 on resting-state functional connectivity between IC-38 and IC-35 was significant (*F* (2, 30958) = 9.69, *p* < 0.001) and remained significant after adjusting for multiple comparison correction (corrected *p* = .034). Finally, as depicted in Fig. 2, post-hoc comparison revealed that the GG genotype at *OXT* rs4813627 (*M* = 0.19, *SD* = 0.59) had lower resting-state functional connectivity (between IC-38 and I-35) than the GA genotype (*M* = 0.23, *SD* = 0.60, *z* = −4.22, *p* < 0.001). The GG genotype (*M* = 0.19, *SD* = 0.59) also had numerically lower resting-state functional connectivity than the AA genotype (*M* = 0.21, *SD* = 0.60), but this effect was not statistically significant (*z* = −1.59, *p* = 0.334). In addition, the GA genotype (*M* = 0.23, *SD* = 0.60) had higher resting-state functional connectivity than the AA genotype (*M* = 0.21, *SD* = 0.60, *z* = 2.58, *p* = 0.030).

**Table 2:** Significant results of ANOVA Testing for Oxytocin Pathway Gene SNPs and Their Impact on Brain Connectivity Components.

Gene	SNP	Component 1	Component 2	*F* value	*p* value
*OXT*	rs4813627	38	35	9.69	<0.001
*OXTR*	rs237851	38	18	7.55	0.001
*OXTR*	rs237851	38	42	6.21	0.002
*OXTR*	rs237851	38	52	5.31	0.005
*OXTR*	rs53576	38	50	5.17	0.006
*OXTR*	rs7632287	38	49	4.87	0.008
*OXTR*	rs7632287	38	50	4.86	0.008
*OXTR*	rs7632287	38	29	4.77	0.008
*OXTR*	rs237887	38	1	4.72	0.009
*OXTR*	rs53576	38	23	4.60	0.010
*OXTR*	rs53576	38	34	4.58	0.010
*OXTR*	rs237851	38	23	4.35	0.013
*OXTR*	rs237851	38	49	3.92	0.020
*OXTR*	rs2268498	38	30	3.92	0.020
*OXTR*	rs2268498	38	27	3.90	0.020
*OXT*	rs4813627	38	48	3.87	0.021
*CD38*	rs3796863	38	19	3.80	0.022
*OXTR*	rs11914885	38	55	3.63	0.027
*OXTR*	rs11914885	38	39	3.62	0.027
*CD38*	rs3796863	38	54	3.62	0.027
*CD38*	rs3796863	38	35	3.59	0.028
*OXTR*	rs2268498	38	50	3.57	0.028
*CD38*	rs3796863	38	8	3.43	0.032
*OXTR*	rs53576	38	41	3.34	0.035
*CD38*	rs3796863	38	50	3.34	0.035
*OXT*	rs4813627	38	43	3.28	0.038
*OXT*	rs4813625	38	29	3.22	0.040
*OXT*	rs4813627	38	10	3.22	0.040
*OXTR*	rs237887	38	17	3.18	0.042
*OXTR*	rs2268498	38	49	3.09	0.046
*OXTR*	rs7634632	38	52	3.07	0.046
*OXT*	rs4813627	38	28	3.03	0.048
*OXTR*	rs53576	38	8	3.01	0.049

To test the robustness of our results, we conducted cross-validation. Using the full dataset, the regression model that survived Bonferroni correction yielded *R*^2^ = 0.0008764 and *RMSE* = 0.5985823, with resting-state functional connectivity between IC-38 and IC-35 as the outcome variable, *OXT* rs4813627 as the fixed factor, sex and age as random factors, and the first 10 genetic principal components as covariates. Next, we performed 10-fold cross-validation, where the dataset was split into 10 equally sized folds. The model was trained on 9 folds and tested on the remaining fold, and this process was repeated 10 times with each fold serving as the test set once. The two metrics averaged over the 10 iterations (*R*^2^ = 0.0004460, *RMSE* = 0.5987999) were very close to those from the full dataset. As shown in Table 3, the *R*^2^ and *RMSE* varied slightly across the 10 iterations, with very small standard deviations for both metrics (*SD* of *R*^2^ = 0.00048, *SD* of *RMSE* = 0.00794). There results demonstrated the high robustness of our findings, as the performance metrics from the cross-validation were consistent with those obtained from the full dataset.

**Table 3:** *R*^2^ and *RMSE* values from each iteration of 10-folder cross-validation for the association between rs4813627 and resting-state functional connectivity between IC-38 and IC-35.

Fold	*R*^2^	*RMSE*
1	0.0003558	0.6128534
2	0.0002502	0.5953909
3	0.0001033	0.5962182
4	0.0001762	0.6007521
5	0.0009067	0.5930055
6	0.0002184	0.5885964
7	0.0002609	0.5998051
8	0.0007941	0.6100970
9	0.0002773	0.6012117
10	0.0001450	0.5900956

## 4 Discussion

This study utilized a large sample from the UK Biobank to shed light on the association between genetic variants within oxytocin pathway genes and resting-state functional connectivity among different brain networks. Results indicated that a corticostriatal circuit, involved in social-cognitive, motivational, and affective processes, was associated with *OXT* rs4813627 variations. In particular, individuals with the *OXT* rs4813627 GG genotype had weaker resting-state functional connectivity in this corticostriatal circuit (between IC-38 and IC-35) compared to allele A carriers (GA/AA genotypes).

Previous evidence on *OXT* rs4813627 is limited, but the existing work has suggested its relationship with social functions. For example, Mileva-Seitz et al. [40] discovered that mothers with the *OXT* rs4813627 GG genotype exhibited shorter periods of instrumental care in response to their infants' activities (crying, reaching toward them, and smiling at them), such as adjusting, grooming, and cleaning their infants, particularly for mothers who themselves had better early care quality. Smarius et al. [41] furthermore reported that children carrying *OXT* rs4813627 GG showed less anxiety at age 5–6 and less emotional symptoms at age 11–12 if they were exposed to maternal verbally aggressive behavior at age 1, compared with carriers of the A allele within this SNP. Thus, the *OXT* rs4813627 GG genotype may be associated with reduced sensitivity to other people's behaviors, which is the core feature of certain neurodevelopmental conditions such as ASD [42,43].

Based on our data, weaker resting-state functional connectivity in the corticostriatal circuit (between IC-38 and IC-35) may contribute to *OXT* rs4813627 GG genotype's social insensitivity. The IC-38 encompasses subcortical regions such as the striatum (putamen and caudate nucleus), thalamus, and pallidum, which are enriched with expression of oxytocin pathway genes [37]. These same regions are also largely part of the reward and motivation subcortical network, which may serve as a functional connectivity hub connecting to several cortical networks related to empathy, embodied simulation, mentalizing, and emotion regulation [44,45]. Interestingly, IC-35 identified in the UK Biobank data comprises the precentral gyrus and extends to the postcentral gyrus, medial frontal gyrus, paracentral lobule, supplementary motor area, and sub-gyral regions that are part of the empathy and mentalizing network [[45], [46], [47]]. Therefore, the corticostriatal functional connectivity between IC-38 and IC-35 may be involved in regulating people's understanding of others' needs, empathizing with others' feelings, and thus responding to others' behaviors. In line with this, mothers who had higher synchrony with their children regarding positive social engagement displayed greater functional connectivity between the reward/motivation subcortical network (overlapping with IC-38) and the empathy/mentalizing network (overlapping with IC-35) [48]. Moreover, recent studies found that compared with their typically developing peers, those with ASD showed weaker corticostriatal functional connectivity [49]. Our finding of the *OXT* rs4813627 GG genotype being associated with weaker corticostriatal resting-state functional connectivity between IC-38 and IC-35 may also partially explain why mothers with the *OXT* rs4813627 GG genotype were less responsive to their infants' activities [50].

One prominent account in the literature proposes that the oxytocinergic system modulates salience of relevant social cues [50]. When exposed to external stressors such as early life adversities, higher functioning of the oxytocinergic system may exaggerate the impact of stressors, resulting in stronger affective responses. In contrast, individuals with lower functioning of the oxytocinergic system may be less susceptible to external stressors [50]. Our finding that the *OXT* rs4813627 GG genotype was associated with weaker corticostriatal resting-state functional connectivity between IC-38 and IC-35 may indicate lower functioning of the oxytocinergic system and help to explain why children with the *OXT* rs4813627 GG genotype were less influenced by their mothers’ aggressive behaviors [41].

The current research tested a group of single SNPs within oxytocin pathway genes. This method is different from the single SNP approach [17] or the polygenic score approach based on results from genome-wide association studies (GWAS) [51], which both focus on a particular phenotype (e.g., depression, autism), often with an aim to test whether a single SNP or a polygenic score of this phenotype could predict (or interact with other variables to predict) the incidence rate of this phenotype. The present study, in contrast, did not investigate genetic differences in a particular phenotype. Instead, we focused on oxytocin pathway genes, which regulate synthesis and release of oxytocin and oxytocin receptors and include many SNPs [15,26,27], and determined their association with functional brain connectivity patterns.

Our study makes several important contributions to the literature. First, unlike previous studies that have typically focused on single *OXTR* SNPs, based on recent reviews and recommendations, we simultaneously considered a large number of relevant SNPs from three well-studied oxytocin pathway genes (i.e., *OXT*, *CD38*, and *OXTR*) [15,26,27]. This approach allows us to test which gene(s) play(s) a particularly important role(s) in regulating resting-state brain connectivity. Second, our findings support the notion that oxytocin directly influences brain activity in regions enriched with expression of oxytocin pathway genes and that this activity then spreads to other regions via intrinsic neural connections [[19], [20], [21]]. The results from our study have the potential to spur future studies that aim to trace oxytocin in the brain, thereby more formally testing its neural mechanism of action. Third, our approach is also unique in the use of a large sample size (with over 30,000 cases from the UK Biobank), which goes beyond the large majority of previous studies [16,26] and allows to draw firmer conclusions (also considering the demonstrated statistical sensitivity to detect even small effects in our sample).

Taken together, our findings generate a novel understanding of associations between genetic variants within oxytocin pathway genes and resting-state functional connectivity. Moving forward, our results have potential to inform the neural mechanisms by which oxytocin-related genetic factors influence social behaviors. However, the present study also has some limitations that should be considered when interpreting the findings. First, false discovery rate is generally high in candidate gene studies [52]. Although our sample was sufficiently sensitive to detect small effects, replication of our findings in an independent sample would confirm robustness of our observations. Second, our analysis was conducted on a white British population from the UK Biobank, limiting our findings' generalizability to other demographics. Furthermore, the UK Biobank sample primarily consists of middle-aged and older individuals (ranging from approximately 45 to 82 years), potentially restricting the applicability of our results to younger age groups. Second, our analysis was conducted on a white British population from the UK Biobank, limiting the generalizability of our findings to other racial/country demographics. Furthermore, the UK Biobank sample primarily consists of middle-aged and older individuals (ranging from approximately 45 to 82 years), and the applicability of our results to younger age groups cannot be addressed with this data. Given evidence of age-related differences in resting-state functional connectivity [22,53], it is possible that the relationship between *OXT* rs4813627 and the corticostriatal connectivity (between IC-38 and IC-35) observed in our study may not apply to younger adults. We acknowledge this limitation and suggest that future research should explore this relationship within and between a wider age range, and including younger individuals, for a more comprehensive developmental perspective. Third, weaker corticostriatal resting-state functional connectivity of the *OXT* rs4813627 GG genotype may be one of the neural mechanisms underlying its association with ASD. Unfortunately, it was not possible for us to test this mediating effect because there were only 30 ASD cases in the dataset. Future investigations will benefit from testing this possibility directly, which has important implications for brain-based intervention. Furthermore, using novel mRNA-based approaches [37] can be leveraged in the future for fine-grained analysis of the molecular mechanisms underlying the associations between variations of oxytocin pathway genes and resting-state functional connectivity, such as how mRNAs of oxytocin pathway genes mediate the observed associations. Lastly, our cross-sectional design can test correlations but not causation. Longitudinal approaches and pharmacological studies using specific treatments to influence the expression of oxytocin pathway genes will shed light on the causal relationships between genetic variations and resting-state functional connectivity for confirmation and extension of the present study's research findings.

## 5 Conclusion

In sum, the present study provides evidence for an association between the *OXT* rs4813627 common genetic variant and resting-state functional connectivity within the corticostriatal circuit based on the analysis of a large, population-based sample from the UK Biobank. The observed association points to a potential neural mechanism through which oxytocin-related genetic factors influence social functions. Our findings underscore the importance of considering genetic contributions to the complex interplay of neural circuits involved in sensitivity to other people's behaviors. Further research is needed to elucidate the precise role of oxytocin pathway genes in shaping brain connectivity and their impact on diverse aspects of social functions, as well as to investigate potential implications of these genetic influences for interventions targeting social deficits broadly as well as in the specific context of relevant neurodevelopmental conditions such as ASD.

## Funding

HF is supported by the 10.13039/501100004359Swedish Research Council (grant Dnr 2013-00854); NCE is partially supported by the 10.13039/100000049National Institute on Aging (grant R01AG059809), the 10.13039/100000026National Institute on Drug Abuse (grant R21DA056813), and the 10.13039/100000006Office of Naval Research (grant N00014-21-1-2201); GR is supported by a grant from the 10.13039/501100000942Swedish Brain Foundation (FO2022-0148); JD is supported by the 10.13039/501100001809National Natural Science Foundation of China (Project 31871098); LW received funding from the 10.13039/501100004359Swedish Research Council (Grant Dnr 2018-02904); HBS is supported by the 10.13039/501100000942Swedish Brain Foundation (FO2023-0090).

## CRediT authorship contribution statement

**Shanshan Xiao:** Writing – review & editing, Writing – original draft, Visualization, Software, Methodology, Formal analysis, Conceptualization. **Håkan Fischer:** Writing – review & editing, Supervision. **Natalie C. Ebner:** Writing – review & editing. **Gull Rukh:** Writing – review & editing, Data curation. **Junhua Dang:** Writing – review & editing, Data curation. **Lars Westberg:** Writing – review & editing. **Helgi B. Schiöth:** Writing – review & editing, Funding acquisition.

## Declaration of competing interest

No conflict.
